# Multi-group Causal Model of Health Literacy and Behaviors on Family Wellbeing among Thai Adults at Risk of Non-Communicable Diseases (NCDs)

**Published:** 2018-09-26

**Authors:** Ungsinun Intarakamhang, Ann Macaskill

**Affiliations:** ^1^ Behavioral Science Research Institute, Srinakharinwirot University, Bangkok, Thailand; ^2^ Psychology Research Group, Faculty of Development and Society, Sheffield Hallam University, Sheffield, United Kingdom

**Keywords:** Psychological factors, Social norm, Healthy behavior, Health literacy, Well-being

## Abstract

**Background:** We aimed to develop a causal model of family well-being with health literacy (HL) as a mediator and to compare models between male and female spouses in urban and rural communities.

** Study design:** A cross-sectional study.

**Methods:** The samples included 2000 spouses at risk of non-communicable diseases (NCDs) by stratified randomly sampled in 2018. Data were collected Likert questionnaires with reliability of 0.79-0.93, and analyzed via confirmatory factor analysis (CFA), and multi-group structural equation modeling (MSEM).

**Results:** A causal model for the overall group was consistent with the data .Causal factors had direct effects on health behavior including social norms, positive attitude toward health, psychological capital, and HL (β=0.11, 0.14, 0.30, and 0.41, *P*<0.05 respectively). Health behavior and positive attitudes towards health had direct effects on family well-being (β=0.36 and 0.42, *P*<0.05, respectively). All factors could predict health behavior and family well-being with variance of 70 %and 50%. Invariance analysis of models showed no difference between male and female spouses. In addition, the mean comparison of latent variables showed that the positive attitudes towards health were lower in women than men. HL and positive attitudes towards health of spouses in urban were lower than in rural communities.

**Conclusion:** Thai adult families in cities were at higher risk with NCDs. Therefore, health providers need to address HL and positive attitude toward health which were the main causal factors.

## Introduction

WHO highlighted concern about the growing incidence of non-communicable diseases (NCDs) worldwide, reporting that globally 70**%** of deaths each year are attributable to NCDs and the main four NCDs are cardiovascular diseases, cancers, chronic respiratory diseases, and diabetes accounting for 80**%** of all NCD deaths^1^.

Developing health literacy (HL) is regarded as core to improving health and well-being. HL is defined as cognitive and social skills which determine the motivation of individuals to access, understand and use information in ways which maintain good health^2^. Individuals with adequate levels of HL displayed more appropriate health behavior and better health outcomes than those with inadequate HL^3,4^. Patients with high HL could control their blood sugar levels 2.03 times better than patients with low HL^5^. Patients' levels of education and disease knowledge influenced their HL and their HL influenced their health behavior^6^. WHO in Shanghai prioritised increasing HL globally^7^. Worldwide surveys of population HL confirmed that it was problematic with low levels found in 32.5% of the population of USA^8^, New Zealand (56.2%)^9^, Bulgaria (62.1%), Spain (58.3%), Austria (56.4%), Germany (46.3%), Greece (44.8%), Poland (44.6%), Ireland (40%), the Netherlands (28.7%)^10^, and Japan (25.3%)^11^. Particularly low HL is reported among the elderly, the poorly educated, indigenous people, and in rural communities^12,13^. In Western cultures, social norms are an important factor influencing health behavior^14^. In an Asian collectivist culture the family plays a role in developing HL^15^. In Thailand, from 2000 to 2014, hypertension prevalence increased fivefold and diabetes by 11%^16^. In 2014, Thais had the second highest rate of obesity in the Association of Southeast Asian Nations^17^. In 2016, a national survey of 15,278 Thai adults found that 49% had low HL, 5.5% high HL, and 63% unhealthy behavior^18^.

The definition of well-being is taken from positive psychology as" a positive state of living, meaning to pursue one's goals, and being satisfied with one's life"^19^.. Character strengths influenced well-being^20,21^. Psychological capital is defined as “an individual’s positive psychological state consisting of self-efficacy, optimism, hope and resilience” ^22^. The pathway model of health guided the choice of variables to be included in the current study^23^. From this model relevant variables were the use of healthcare services, having healthy behavior, self-monitoring, attitudes, social norms, selfefficacy, and HL. The Individual and Family Selfmanagement Theory (IFSM) which specifies the causes of health outcomes and well-being guided the choice of variables such as self-efficacy, social support, community culture, and family and individual context variables^24^.

The aims of the study were to develop a causal model of family well-being and to compare models by moderating between male and female spouses in urban and rural areas.

## Methods

Data for this cross-sectional study were collected via a survey designed to assess the variables identified in the literature review as being relevant to HL and health behaviour. The Thai adult participants between 35-59 year old were selected through a quota-stratified random sampling technique to make sure that both women and men from both urban or semi-urban and rural communities near the country's borders were selected equally. A previous survey^18^ identified ten provinces covering the north, south, northeast, and central Thailand where levels of HL were low and risks of NCDs were high. Data were used to randomly select 200 people from each province via the public health database, giving a total sample of 2000 adults, equally divided between rural and urban areas and men and women. Data were collected between Jan and Apr 2018. The sample size was based on the size required to confirm a causal model, between 100 and 200 people in each group^25^. The research assistants contacted participants by asking the Village Health Volunteers (VHVs) to set up times to meet with them in their own homes.

The survey was administered by four well-trained research assistants to ensure that meaningful data could be collected even if literacy levels were low or if there were difficulties in understanding any of the questions. Husbands and wives completed the survey separately

The Thai Adults Health Questionnaire was developed as a culturally appropriate measure of HL and health behavior .The HL elements were based on the General Health Literacy Scale^13^ developed with an Australian population with a very well-developed health care system so was not totally applicable to the Thai context. This scale was modified to assure cultural relevance in Thailand^18^ .For this study, it was expanded further to make a more comprehensive assessment of HL, health status and associated health behavior. These additional components were informed by reviewing the literature on HL models^13,23^

The questionnaire began with demographic information relating to age, gender, educational level, primary employment, years married or cohabiting, record of any medical problems, and checklist of symptoms relating to NCDs. Next competency in dealing with health-related issues, perceived ease of access to information and services, verification processes used for knowledge and services, communication, management of own and family health, availability of social support, social norms including health-related cultural wisdom and their influence, family role models, attitudes towards health-related behaviours and an assessment of the well-being of the family. All items were rated on a five-point Likert scale and scores were summated.

The questionnaire was assessed for breadth and relevance of content and cultural match by five experts in the field of health behavior, and psychology with high levels of agreement reached. The content analysis of items with index of item-objective congruence (IOC) were between 0.80-1.00. The internal reliability of the questionnaire was satisfactory with Cronbach’s alpha values ≤0.70 and the construct validity by the second confirmatory factor analysis (CFA) was satisfactory with factor loading of items ≤ 0.40^26^.

Pre-analysis checks were carried out on the data set on missing data, outliers, linearity, skewness, kurtosis, *P*>0.05 and multivariate normality^25^ and no problems were found. Demographic descriptive data was computed for all the variables. Then Confirmatory factor analysis (CFA) and multi-group structural equation modeling (MSEM) were computed to test the generated causal relationship model's applicability and to compare the latent variable mean by using LISREL program. Moreover, statistical values included the absolute fit index, Chi-Square (χ^2^) Goodness of Fit Index: GFI ≤0.90, Root Mean Squared Error Approximation: RMSEA ≥0.05, SRMR ≥ 0.05, NNFI, GFI ≤0.90 [Incremental fit index], and adjusted goodness of Fit Index: AGFI ≤0.90, and χ^2^/df ≤5. [Parsimony fit indices]^26^

Documented assent was obtained from all participants who could not provide written consent. The study was approved by the IRB of Srinakharinwirot University (Certificate of approval No. SWUEC/E-264/2560).

## Results

The mean age of the rural sample was males =47.04, SD=7.56; females=45.99, SD=7.45, and the urban sample was males =48.45, SD=7.37; females=47.00, SD=7.17. The modal values for occupational group was farmers (33.8%), for education was elementary education (54.5%), and the duration of living together was 21–25yrs (18.7%). In terms of health risk for NCDs, 75.6% of the sample were not exercising and were overweight. High levels of health literacy were reported in 26% of the sample while 58.5% had levels rated as being inadequate but good levels of family well-being were reported by 61.0% of the sample. The demographics for each sample are shown in Table 1. The mean and standard derivation for each variable were computed. Cronbach's alphas were calculated for each scale and subscale and were satisfactory between 0.77-0.94. The construct validity by CFA with factor loading of items were between 0.45 and 0.87 (Table 2).

**Table 1:** Comparison of Sample demographics between the rural and urban populations and men and women

**Demographics**	**Men, n=1000**		**Women, n=1000**		**Urban, n=1000**		**Rural, n=1000**	
	n	%	n	%	n	%	n	%
Age (yr)								
35-40	214	21.4	266	26.6	212	21.2	268	26.8
41-45	168	16.8	185	18.5	179	17.9	174	17.4
46-50	198	19.8	204	20.4	197	19.7	205	20.5
51-55	216	21.6	211	21.1	223	22.3	204	20.4
56-59	204	20.4	134	13.4	189	18.9	149	14.9
Educational level								
None	47	4.7	42	4.2	25	2.5	64	6.4
Elementary	551	55.1	539	53.9	526	52.6	564	56.4
Middle school	125	12.5	150	15.0	133	13.3	142	14.2
High school or certificate	157	15.7	124	12.4	149	14.9	132	13.2
Associate degree	50	5.0	51	5.1	58	5.8	43	4.3
Bachelor's degree or higher	70	7.0	94	9.4	109	10.9	55	5.5
Occupation								
Agricultural	363	36.3	314	31.4	325	32.5	352	35.2
Shopkeeper	219	21.9	223	22.3	115	11.5	327	32.7
Government official	82	8.2	85	8.5	88	8.8	79	7.9
Employee or workers	105	10.5	117	11.7	163	16.3	59	5.9
Not in paid employment	231	23.1	261	26.1	309	30.9	183	18.3
Time couples married/cohabiting (yr)								
0-5	84	8.4	78	7.8	49	4.9	113	11.3
6-10	109	10.9	112	11.2	89	8.9	132	13.2
11-15	122	12.2	137	13.7	138	13.8	121	12.1
16-20	145	14.5	151	15.1	168	16.8	128	12.8
21-25	190	19.0	184	18.4	186	18.6	188	18.8
26-30	181	18.1	168	16.8	207	20.7	142	14.2
>30	169	16.9	170	17.0	163	16.3	176	17.6

**Table 2:** Means and standards deviation of each latent variable and its constituent scales and Cronbach's alphas for each scale

**Variables**	**Rural**		**Urban**		**Cronbach’s α**	**Factor loading**
	**Mean**	**SD**	**Mean**	**SD**		
Latent variable; HL was measured by 5observable variables
Access to health information and services	3.64	0.81	3.76	0.69	0.82	0.46-0.75
Understanding of health information and services	3.59	0.86	3.76	0.73	0.83	0.51-0.80
Verification of health information and services	3.73	0.73	3.78	0.72	0.77	0.57-0.78
Communication skill	3.78	0.73	3.75	0.70	0.89	0.49-0.78
Self-health management	3.30	0.70	3.24	0.65	0.79	0.66-0.78
Social support	3.81	0.75	3.81	0.70	0.89	0.61-0.83
Social norms	3.87	0.72	3.69	0.69	0.83	0.66-0.87
Positive attitude toward health	3.77	0.72	3.74	0.63	0.84	0.58-0.84
Psychological capital was measured by 4 observable variables
Hope	3.91	0.76	3.88	0.72	0.93	0.72-0.79
Optimism	3.89	0.70	3.91	0.73	0.94	0.65-0.86
Self-efficacy	3.82	0.75	3.81	0.70	0.93	0.60-0.81
Resilience	3.95	0.76	3.95	0.69	0.93	0.70-0.80
Health behavior was measured by 2 observable variables
Self-care	3.47	0.89	3.69	0.71	0.87	0.45-0.79
Participation in health activities	3.55	0.90	3.68	0.89	0.87	0.75-0.75
Family well-being was measured by 3 observable variables
Health status of family members	4.07	0.75	4.08	0.69	0.89	0.65-0.77
Parents' integrity	4.12	0.82	4.10	0.76	0.89	0.67-0.85
Family relationships	4.08	0.82	4.16	0.80	0.88	0.77-0.86

**Figure 1:**
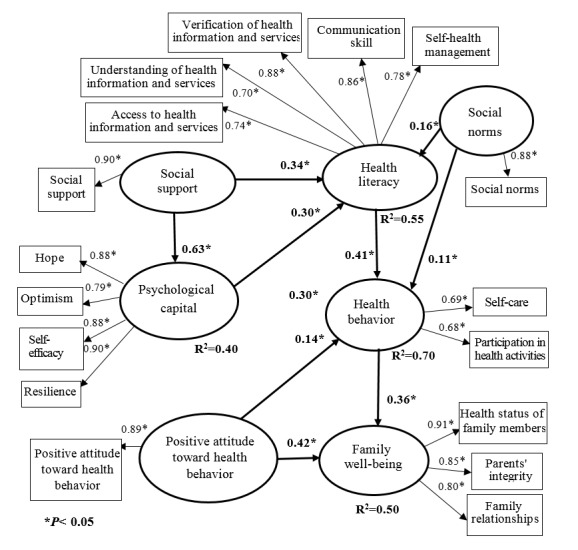
Causal relationship model of social norm and psychological instead capital effects on health behavior and family well-being by mediating HL of spouses at risk of NCDs, in overall group (n=2000)

The results of the hypothesis testing with empirical data showed that the influence and test statistical significance were not significant. The researchers adjusted the model by allowing tolerances to measure if the variables were related. The adjusted model results were as follows:

1) Testing the adjusted model A causal relationship model of social norm and psychology capital affecting health behavior and family well-being by mediating HL in overall group was consistent with the empirical data ꭓ^2^ =228.57, df =67 (*P*=0.00), ꭓ^2^ /df =3.41, RMSEA =0.03, SRMR = 0.02, GFI =0.99, NNFI =99, and AGFI =0.97. In addition, causal factors had direct effects on health behavior including social norm, positive attitudes, psychology capital, and HL (β=0.11, 0.14, 0.30, and 0.41, *P*<0.05 respectively). Besides, health behavior, and positive attitudes had direct effects on family well-being (β=0.36 and 0.42, *P*<0.05 respectively). Total causal variables had indirect effects on family wellbeing such as psychology capital, social support, HL, social norm, and positive attitudes were 0.16, 0.15, 0.15, 0.06, and 0.05, *P*<0.05 respectively. All factors could predict health behavior and family well-being with variance of 70% and 50% (Figure 1 and Table 3).

**Table 3:** Influence coefficient (β) in the adjusted model effected on health behavior and family well-being by mediating HL in overall group

**Causal variables**	**Psychological capital** **(R2= 0.40)**			**Health literacy** **(R2= 0.55)**			**Health behavior** **(R2= 0.70)**			**Family well-being** **(R2= 0.50)**		
	**DE**	**IE**	**TE**	**DE**	**IE**	**TE**	**DE**	**IE**	**TE**	**DE**	**IE**	**TE**
Social support	0.63	0.00	0.63	0.34	0.19	0.53	0.00	0.41	0.41	0.00	0.15	0.15
Social norms	0.00	0.00	0.00	0.16	0.00	0.16	0.11	0.07	0.18	0.00	0.06	0.06
Psychological capital	0.00	0.00	0.00	0.30	0.00	0.30	0.30	0.13	0.43	0.00	0.16	0.16
Health literacy	0.00	0.00	0.00	0.00	0.00	0.00	0.41	0.00	0.41	0.00	0.15	0.15
Positive attitude toward health	0.00	0.00	0.00	0.00	0.00	0.00	0.14	0.00	0.14	0.42	0.05	0.47
Health behavior	0.00	0.00	0.00	0.00	0.00	0.00	0.00	0.00	0.00	0.36	0.00	0.36

DE = Direct effect, IE = Indirect effect, TE =Total effect

**Figure 2:**
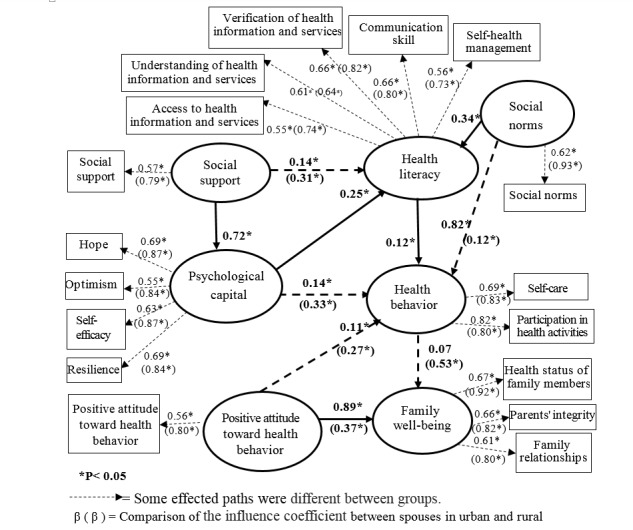
Results of influence coefficient estimation in when comparing the causal relationship model affecting health behavior and family well-being by mediating HL between spouses in urban (n=1000) and rural (n=1000)

2) Differences in responding in the model between male and female spouses and between urban and rural respondents were examined by testing the invariance of causal models and comparing the means of the latent variables. The results indicated no differences in the causal models between men and women (Δc^2^= 13.22, Δ df= 10, *P-*value= 0.21) In terms of the direct and indirect influences of the causal factors on health behavior, and family well-being there were no difference between male and female spouses. There were statistically significant differences in the causal models between spouses in the urban and rural communities (Δc^2^= 93.31, Δdf= 10, *P*=0.001). These differences in effect size and factor loading on some of the paths in the causal model are shown in Figure 2.

3) Comparison of the means of the latent variables showed that positive attitudes toward health in women was lower than in men (d= 0.06, SE= 0.03, t-value= 2.08, *P*<0.05). There were no significant differences in the mean scores between men and women for social support, social norms, psychological capital, HL, health behavior and family well-being (Table 4). There were significant difference in mean scores HL (d= 0.11, SE= 0.02, *t*-value=5.64), positive attitudes toward health (d= 0.10, SE= 0.03, *t*-value= 3.34), and family well-being (d= -0.09, SE= 0.03, *t-*value= 2.93) of spouses were lower in urban than rural communities (Table 5).

**Table 4:** Comparison of the latent variable average in the causal relationship model affecting to health behavior and family well-being by mediating HL between male and female spouses

**Latent variables**	**Average difference (d)**	**Standard error (SE)**	***t*** **value**
Social support	0.05	0.03	1.90
Social norms	0.04	0.03	1.46
Psychological capital	0.02	0.03	0.82
Health literacy	0.01	0.02	0.70
Positive attitude toward health	0.06	0.03	2.08^*^
Health behavior	0.02	0.03	0.75
Family well-being	0.02	0.03	0.68

^*^ *P*<0.05, Average difference= Mean of latent variable in men- Mean in women group

**Table 5:** Comparison of the latent variable average in causal relationship model affecting to health behavior and family well-being by mediating HL between spouses in urban and rural communities

**Latent variables**	**Average difference (d)**	**Standard error (SE)**	***t*** **value**
Social support	0.00	0.03	-0.01
Social norms	-0.02	0.03	-0.67
Psychological capital	0.00	0.02	0.17
Health literacy	0.11	0.02	5.64^*^
Positive attitude toward health	0.10	0.03	3.34^*^
Health behavior	0.03	0.03	1.03
Family well-being	0.09	0.03	2.93^*^

^*^ *P*<0.05, Average difference=Mean of latent variable in rural - urban spouses

## Discussion

We found that all factors could significantly predict health behavior, and family well-being of Thai adult families in these communities. According to the logic model^23^ and systems theory that predict health behavior and health outcome well-being increased with higher health literacy^24^. In this model for the overall group, 70% of health behavior and 50% of family well-being could be explained by the all factors. HL had highest direct influence on health behavior, psychology capital, positive attitudes, and social norm, respectively. Both men and women with higher HL, psychology capital, positive attitudes, and social norm reported better health. They participated in health activities and maintained self-care in higher level too. HL directly influenced the health behavior of diabetic patients^5^. Critically, HL influenced obesity preventive behaviors, and motivation, with functional HL associated with diet in type 2 diabetics^27^. Including, health literacy development, the activities focused on ways of searching for correct health information, health information access skills, using social media safely, and exchanging health information to improve self-care behavior and encourage patients to self-care^28^. Additionally, health literacy had indirect effect on well-being measured by participating in social activities^18^. Health knowledge and understanding had an indirect effect on participating in social activities via mediation managing their health condition, media literacy, appropriate decision-making, and maintaining in health behaviors.

Psychological capital affected directly health behavior and indirectly family well-being (β =0.30, 0.16 respectively). Psychological capital refers to an individual's positive psychological strengths which lead to behavior change^22^. Therefore, psychological capital represents an individual's positive characteristics such as self-efficacy, hope, optimism, and resilience developed and used to motivate an individual to work effectively. Developing psychological capital such as hope, efficacy, resilience, and optimism in college students significantly increased their positive health^29^. This supports a German study that highlighted the association between psychological factors (Positive and negative affect, life satisfaction, optimism, self-esteem, self-efficacy, and self-regulation) and frequent attendance in primary care of 7,446 people with aged 40. This is consistent with similar research in Thailand^30^.

Positive attitudes toward health and social norms influenced health behavior. The theory of planned behavior was strongly supported in this study with positive attitudes and subjective norm as predictors of health behavior^14^. These findings are supported by a study of an educational intervention on attitude, subjective norms, parental control, and behavioral control31 that produced significant reductions in risky sexual and reproductive health behavior. Social support directly influenced HL in the study. Similarly, social support was the strongest predictor of interactive and critical health literacy of 650 Chinese students aged 7-9 yr^32^. Social support is an important health variable previously shown to improve the psychosocial health status of soldiers and reduce their levels of depression^33^. Social support similarly predicted lower depression in the elderly in a community study^31^. The presence of social support and the introduction of activities to generate such support are important to improve health and wellbeing.

Comparisons of the effect sizes and latent variable averages in the causal relationship models by gender were not significant, nor were the invariance models. Factors such as social support, social norms, psychological capital, and positive attitude toward health influence health behavior and family well-being by mediating health literacy, This applies for, men, women and the overall group. Thus, gender had no interaction with any latent variables in this model. Hence, common activities addressing the same variables can be implemented for men and women to promote healthy behavior and well-being. While, no differences in the causal models was found between spouses in the urban and rural areas and HL; positive attitudes toward health and family well-being were lower in urban spouses than in rural communities. Many rural areas of Thailand have recently changed to become urban areas. However, medical provision in these areas has not been fully developed yet. This may help to explain the lower scores in these recently urbanized areas compared with the rural areas where services are more established. This result is inconsistent with a population surveys of Chinese adults where the prevalence of metabolic syndrome (MS) whith raised fasting glucose and raised blood pressure was significantly higher in rural residents than in their urban counterparts. They looked, at newly urbanized Chinese areas similar to those found in this study and found that as urbanization progressed and health services were introduced health improved in theses previously rural areas^34^. This demonstrates the impact of community health care services. In Thailand rural health care is well developed but is poorer in the more recently urbanized areas. Health providers need to be aware of the urgent need to provide local health care services as urban areas expand to support the development of health literacy and promote wellbeing in theses urbanised communities. While the urban or semi-urban and rural communities share features such as the prevalence of NCDs and socio-psychological risk factors, the differences found may be partly explained by lifestyle factors and the population changes as people move to urban areas. Given the high prevalence of NCDs in Thai adults intervention strategies are required to address these rural-urban disparities.

## Conclusion

The majority of the Thai adult families had health risks by not exercising, being overweight, and having inadequate HL levels. All factors; psychology capital, social support, HL, social norms, and positive attitudes predicted health behavior. Moreover, good health behavior and positive attitude toward health had positive direct effects on family well-being. There was no difference in the causal relationship model of HL and family well-being between male and female spouses although women had less positive attitudes towards health than men.

## Acknowledgements

The authors thank all participants include the health provider as research assistants and the chief of medical officers as gatekeeper of the target communities in this study.

## Conflict of interest statement

This work is original and has not been published elsewhere nor is it currently under consideration for publication elsewhere. The authors declare no conflict of interest.

## Funding

This study, as a part of multi-phase project, was supported the Newton Advanced Fellowships, ﬁnancially supported via the British Academy (Grant No. AF170002/2017), United Kingdom and Thailand Research Fund (DBG61/2561).

## Highlights

- Health literacy and attitude are key factors affecting health behaviors and well-being.
- Health literacy and character development improve well-being sustainably.
- Health providers should use these results to designing NCDs prevention interventions.
